# Assessment of Antidepressant-like, Anxiolytic Effects and Impact on Memory of *Pimpinella anisum* L. Total Extract on Swiss Albino Mice

**DOI:** 10.3390/plants10081573

**Published:** 2021-07-30

**Authors:** Imane Es-safi, Hamza Mechchate, Amal Amaghnouje, Amine Elbouzidi, Mohamed Bouhrim, Noureddine Bencheikh, Christophe Hano, Dalila Bousta

**Affiliations:** 1 Laboratory of Biotechnology, Environment, Agrifood, and Health, Faculty of Sciences Dhar el Mahraz, University of Sidi Mohamed Ben Abdellah, Fez 30050, Morocco; hamza.mechchate@usmba.ac.ma (H.M.); amal.amaghnouje@usmba.ac.ma (A.A.); bousta.dalila@usmba.ac.ma (D.B.); 2 Laboratory of Bioresources, Biotechnology, Ethnopharmacology, and Health, Faculty of Sciences, Mohammed First University, Oujda 60040, Morocco; amine.elbouzidi@ump.ac.ma (A.E.); mohamed.bouhrim@gmail.com (M.B.); bencheikh_noureddine1718@ump.ac.ma (N.B.); 3 Laboratoire de Biologie des Ligneux et des Grandes Cultures, INRAE USC1328, University of Orleans, CEDEX 2, 45067 Orléans, France; hano@univ-orleans.fr

**Keywords:** depression, anxiety, anise seeds, Tail Suspension Test (TST), Forced Swimming Test (FST), Open Field Test (OFT), Light–Dark Box Test (LDBT), Novel Object Recognition Test (NORT), Morris Water Maze Test (MWMT)

## Abstract

Mental disorders are psychological symptoms that impact multiple areas of an individual’s life. Depression and anxiety are chronic illnesses described as the most prevalent stress-related mood disorders that cause injury and early death. In Morocco, Anise “*Pimpinella anisum* L.” is one of the most traditionally used condiment plants, which has long been used to cure various illnesses and in phytotherapy. The present study was designed to investigate the antidepressant, anxiolytic, and memory impact of the total extract of *Pimpinella anisum* (PATE) at the doses of 100 and 200 mg/kg, using the Forced Swimming Test (FST), Tail Suspension Test (TST), Open Field Test (OFT), and Light–Dark Box Test (LDBT) as an experimental paradigm of anxiety and depression, and Novel Object Recognition Test (NORT) and the Morris Water Maze Test (MWMT) as memory tests on Swiss albino mice. The tests were carried out on the 1st, 7th, 14th, and the 21st days of the study, and the extract groups were compared with normal controls and positive controls (receiving bromazepam and paroxetine at the doses of 1 mg/kg and 11.5 mg/kg for anxiety and depression, respectively). The daily oral gavage of the mice by the PATE induced a significant anxiolytic and antidepressant-like effect by shortening immobility time and decreasing downtime in the different tests. PATE at both doses was shown to have no impact on memory following the NORT and MWM tests. Different compounds, such as gallic acid, catechin, chlorogenic acid, caffeic acid, oleuropein, *p*-coumaric acid, trans-4-hydroxy-3-methoxycinnamic acid, myricetin, and quercetin, were identified during the phytochemical analysis carried out using HPLC analysis. This research supports and promotes the extract’s traditional use, suggesting its use as a phytomedicine against depression and anxiety, and calls for further research to clarify its mode of action.

## 1. Introduction

Depression and anxiety have become two of the most prevalent and common mental disorders worldwide and have become a major issue in our societies where stress is omnipresent [1]. On a global scale, about 300 million individuals are estimated to suffer from depression [2]. Anxiety is a physiological response that serves to protect the organism when it faces danger. When it is severe and/or chronic, it becomes pathological and could promote the development of cardiovascular and/or psychological disorders [3]. The treatment of anxiety involves behavioral therapies and drugs such as benzodiazepines and serotonin reuptake inhibitors [4]. Depression is considered one of the leading causes of disability globally, having a very significant impact on morbidity, mortality, and leading in some cases to suicide. According to the World Health Organization (WHO), it is considered a mental disorder, the third leading cause of comorbidity in the world, and will be the second leading cause of death and disability by 2021 [5]. The proposed treatment is antidepressants such as Imipramine, Desipramine, Amoxapine (tricyclic antidepressants (TCAs)) and serotonin reuptake inhibitors. These drugs target mainly monoamine and serotonin transporters [6]. Depression and anxiety treatments only act in the long term. They are fraught with side effects, such as orthostatic hypotension, tachycardia, digestive disorders, weight gain, sexual disorders, or even visual disorders, and one major problem that comes after more prolonged exposure is memory impairment [7,8]. It has been declared that long-time use of benzodiazepines affects memory [9].

Benzodiazepines are attached to a particular receptor and promote the action of GABA on the receptor of a neurotransmitter in the brain. GABA lowers central nervous system excitability, and this reduction can have many beneficial consequences, as demonstrated by their numerous applications. Benzodiazepines are often used to relieve anxiety, sleep disturbances, and a variety of other medical conditions. Nevertheless, harmful consequences are gradually becoming recognized [4]. Some benzodiazepines are correlated to some severe central nervous system (CNS) and psychiatric problems associated with memory loss and cognitive decline [8].

For this reason, new therapeutic avenues are emerging in favor of natural substances [10]. Nowadays, herbal treatments are coming to the forefront because these remedies are natural, safe, and present an undeniable efficacy against different health problems, such as cardiovascular problems, diabetes, and cancer, and they are included in the composition of several drugs [11,12]. Among these medicinal plants, we investigate the potential anti-depressive and anxiolytic effects of anise (*P. anisum*), a species of herbaceous plant in the Apiaceae family, a flowering plant (30 to 50 cm tall) with white flowers, cultivated as a condiment plant mainly for its aromatic leaves and seeds [13]. This plant is often called green anise, originating from the Eastern Mediterranean region and Southwest Asia. The seeds of *P. anisum* are known for their numerous biological activities beneficial for human health [14,15]. They have diuretic, antihypertensive, antidiabetic, anticancer, immunomodulating, antimicrobial, analgesic, anti-inflammatory, antioxidant, and anti-stress effects [16]. This study was conducted to evaluate the antidepressant, anxiolytic, and memory impact of the total extract of *P. anisum* using these tests: the Forced Swimming Test (FST) and the Tail Suspension Test (TST); the Open Field Test (OFT) and the Light–Dark Box Test (LDBT); the novel object recognition test (NORT) and the Morris maze test (MWT), respectively, on Swiss albino mice. The extract was characterized using the HPLC-UV technique.

## 2. Results

### 2.1. Identification of the Major Constituents of the Seed Extract

**Figure 1:**
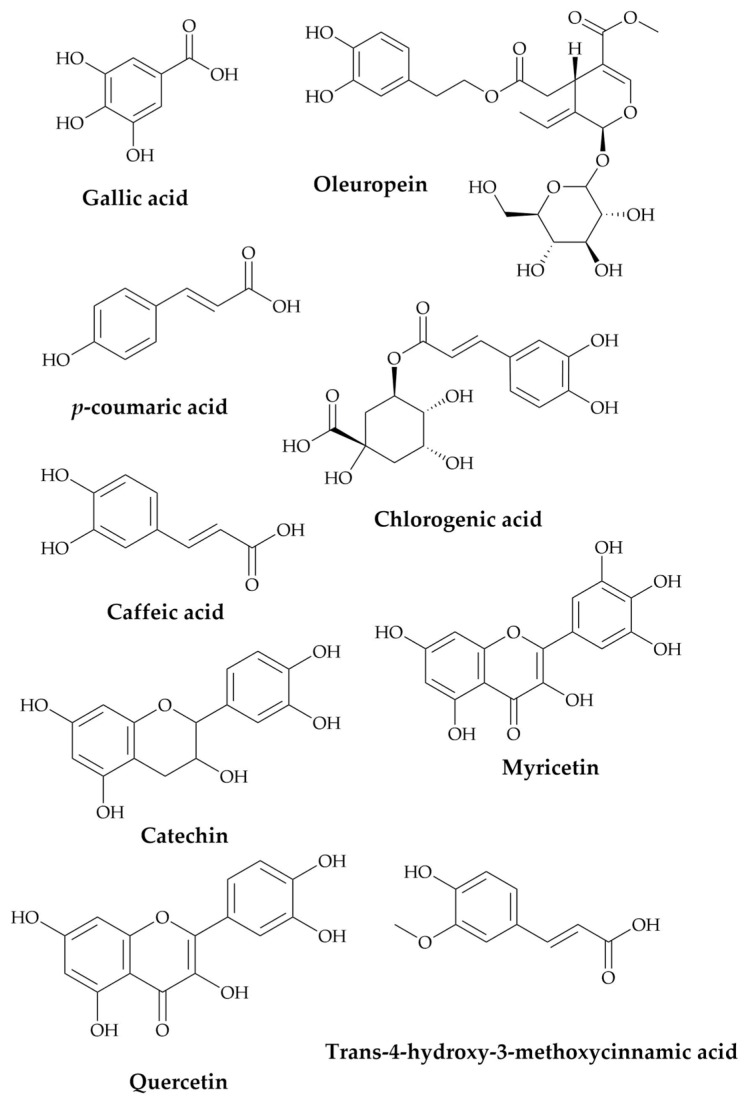
Chemical structures of PATE principle bioactive components.

PATE phytochemicals were revealed using HPLC (see chromatogram in the supplementary file), and the details of the bioactive components and their chemical structures are shown in Table 1 and Figure 1. The most abundant compounds in the extract were *p*-coumaric acid and catechin, followed by oleuropein, gallic acid, caffeic acid, chlorogenic acid, trans-4-hydroxy-3-methoxycinnamic acid, quercetin, and myricetin.

### 2.2. Evaluation of the Antidepressant Activity

#### 2.2.1. Forced Swimming Test

**Figure 2:**
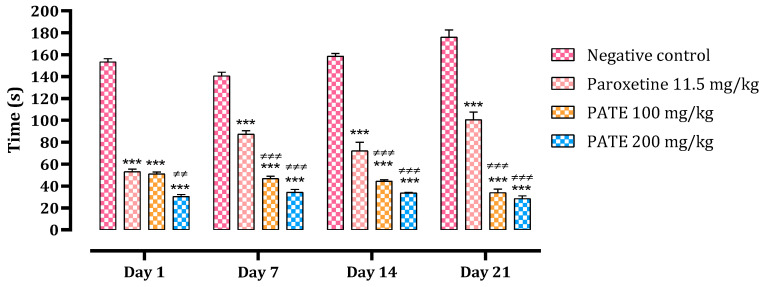
The effect of PATE on the immobility time variation in mice. Data are represented as mean ± SEM. (*** *p* ≤ 0.001 compared to negative control, ≠≠ *p* ≤ 0.01, ≠≠≠ *p* ≤ 0.001 compared to positive control).

The difference in immobility time in control mice and mice treated with 100 and 200 mg/kg of PATE is shown in Figure 2. On day one, different treatments administered significantly reduced the immobility time compared to the normal control (by 67% with the dose of 100 mg/kg, 80% with the dose of 200 mg/kg, and 65% with paroxetine). This reduction continued until day 21, with 81% noted with the dose of 100 mg/kg, 84% with the dose of 200 mg/kg, and 43% with paroxetine compared to the normal control. Both PATE doses were more effective than that of paroxetine, especially at the end of the test. The extract efficiency was time-dependent.

#### 2.2.2. Tail Suspension Test

**Figure 3:**
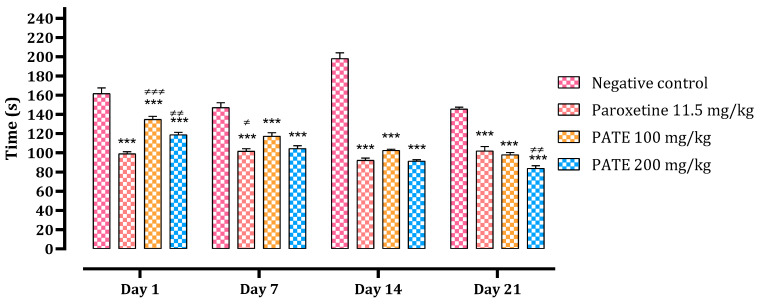
Downtime of the animals in TST. Data are represented as mean ± SEM. (*** *p* ≤ 0.001 compared to the negative control group. ≠ *p* ≤ 0.05, ≠≠ *p* ≤ 0.01, ≠≠≠ *p* ≤ 0.001 compared to the paroxetine group).

The immobility time of the mice in the tail suspension was recorded during 6 min of testing. On day 1, different PATE treatments administered slightly reduced the immobility time compared to the normal control (by 17% with the dose of 100 mg/kg, 27% with the dose of 200 mg/kg), while it was significantly (*p* ≤ 0.001) reduced in the group treated with paroxetine (38%). After 21 days, both PATE treatments noted a significant reduction (with 32% and 42% for the dose of 100 and 200, respectively). The extract results (immobility time) reached those for paroxetine at the end of the 21 days of treatment (92.33 ± 2.17, 102.5 ± 1.258 and 91.33 ± 1.542 s for paroxetine, PATE 100 mg/kg, and PATE 200 mg/kg, respectively). The effect of the extract was as observed time-dependent (Figure 3).

### 2.3. Evaluation of the Anxiolytic Activity

#### 2.3.1. Light Dark Box Test

The administration of PATE by the oral route in mice increases their time spent in the light compartment compared to the mice of the control group as an indicator of an anxiolytic effect.

The different treatments at all study periods increased the time spent in the lighter area, especially for the group treated with bromazepam which increased by 94%, against 9% and 62% for the doses of 100 and 200 mg/kg of PATE, compared to the normal control.

**Figure 4:**
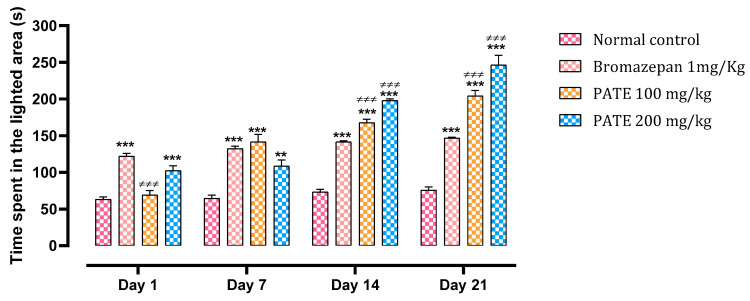
Effect of PATE on the variation in time spent in the lighted chamber in mice. Data are represented as mean ± SEM. (*** *p* ≤ 0.001 compared to the negative control. ≠≠≠ *p* ≤ 0.001 compared to the positive control).

Continuous administration of PATE at both doses demonstrated a higher significant effect of *p* ≤ 0.001 at the end of the test, surpassing that of bromazepam, with 172% noted with the dose of 100 mg/kg and 228% noted with the dose of 200 mg/kg, against 96% noted for bromazepam compared to the normal control. During the study period, the group treated with the extract demonstrated both dose- and time-dependent effects, with the dose of 200 mg/kg being the most efficient (Figure 4).

#### 2.3.2. Open Field Test

**Figure 5:**
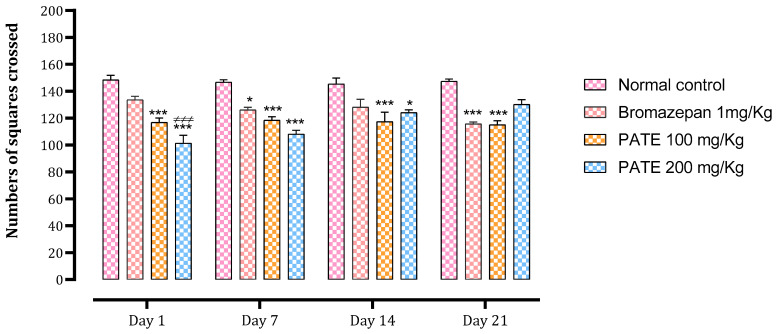
Effect of PATE on the variation in the number of tiles traversed during OFT in mice. Data are represented as mean ± SEM. (* *p* ≤ 0.05, *** *p* ≤ 0.001 compared to the negative control. ≠≠≠ *p* ≤ 0.001 compared to the positive control).

The number of total squares crossed and the time spent in the center were used as indices to assess the anxiolytic effect of the treatments. The bromazepam and PATE groups significantly decreased the number of total squares crossed after the seventh day of treatment (*p* ≤ 0.001) till the end of the 21 days of the study period (Figure 5).

**Figure 6:**
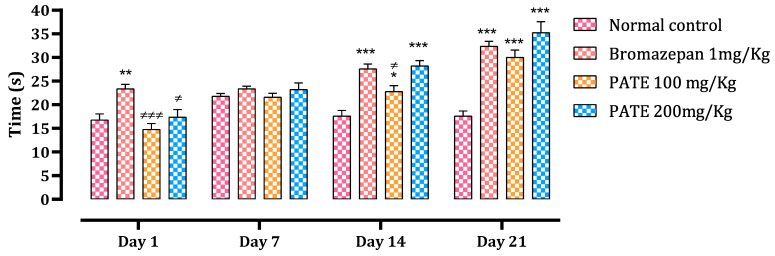
Effect of PATE on time spent variation at the center of the open field in mice. Data are represented as mean ± SEM. (* *p* ≤ 0.05, ** *p* ≤ 0.01, *** *p* ≤ 0.001 compared to the normal control. ≠ *p* ≤ 0.05, ≠≠≠ *p* ≤ 0.001) compared to the positive control.

Throughout all times of the study, data analysis of time spent in the center showed a significant difference between the normal control and the treated groups. Animals treated with PATE demonstrated a time-dependent effect that was compared to the bromazepam at the end of the test (Figure 6).

### 2.4. Memory Impact Evaluation of PATE

#### 2.4.1. Morris Water Maze Test

**Figure 7:**
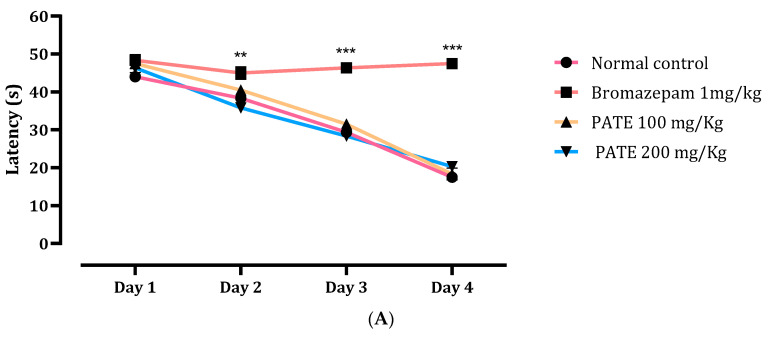

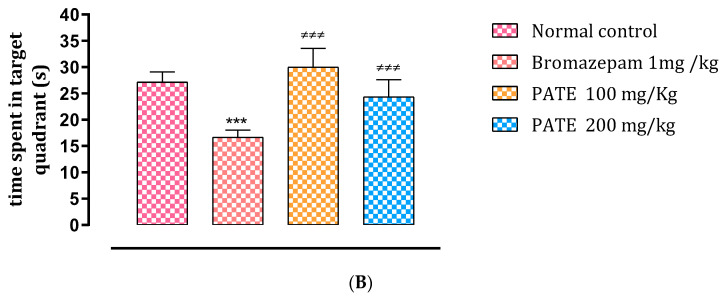
Effects of PATE in the MWMT. (**A**) Latency time and (**B**) time spent in the target quadrant. Data are represented as mean ± SEM. (** *p* ≤ 0.01, *** *p* ≤ 0.001 compared to the normal control group. ≠≠≠ *p* ≤ 0.001 compared to the positive control).

The results of the time latency to find the target quadrant demonstrated a large difference between the first, second, third, and fourth days, as per the analysis. The latency time of mice treated with PATE (100 and 200 mg/kg) and the normal control was reduced between the first and fourth day, while there was not a significant difference throughout the latency time of bromazepam all through the testing period (Figure 7A).

On the fifth day, the main quadrant was noted in comparison to the other quadrants as a recovery memory index. Following the results, the group treated with bromazepam spent significantly less time in the correct quadrant than the normal group, while the group treated with 200 mg/kg spent significantly more time in the correct quadrant. Those results confirm the side effects of the conventional drugs on memory (Figure 7B).

#### 2.4.2. Novel Object Recognition Test

To test the ability of the mice to recognize novel and familiar objects, this test was performed. The test results were presented as a discrimination index (DI) to assess the ability of mice to recognize the familiar object and spent more time to discover the novel one. The test results are analyzed as follows: a negative DI indicates that the mice did not properly recognize the familiar object and tend to rediscover it again, while a positive DI value indicates that the mice recognize the familiar object and then spent more time discovering the novel one. As seen in the results, continuous administration of bromazepam for 21 days altered the mice’s memory ability to recognize the familiar object compared to other groups treated with the extract or the normal control.

**Figure 8:**
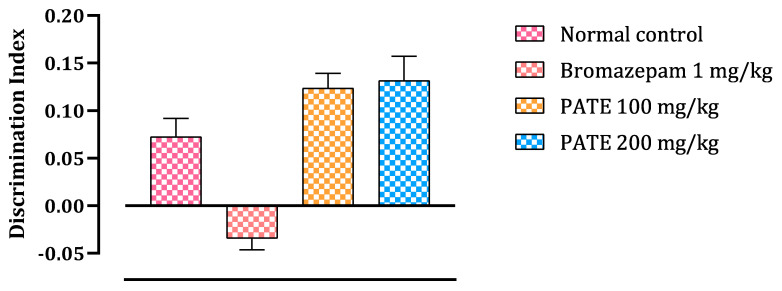
Discrimination index in the NORT. Values are expressed as mean ± SEM.

The bromazepam group spends less time investigating the various objects during the training phase than the PATE groups (100 and 200 mg/kg), which spend more time than the normal group. The two PATE groups (100 and 200 mg/kg) spent more time investigating the new object during the test period, while bromazepam administration decreased the amount of time spent by the mice exploring new objects (Figure 8).

## 3. Discussion

Mental distress is one of the most prevalent types of condition that occurs as a result of a mental health issues and is marked by a variety of complaints, such as sadness, worry, tenseness, or rage [17]. Anxiety, depression, and somatoform conditions are examples of common mental disorders that can impact people all over the world [18,19]. Anxiety and depression affect about 300 million (4.4 percent) and 264 million (3.6 percent) people of the world’s population, respectively, according to a World Health Organization 2015 report [5], and they have been two of the most prevalent forms of disease globally for several years [20]. In accordance with the concern of their rising prevalence, the side effects observed and confirmed for the prolonged use of conventional drugs has been a major dilemma for the authorities that approve such medication with very serious side effects, and also for the patient that has to accept being exposed to those side effects with no other alternative [7]. All those facts combined have stepped up the hunt for other safe and affordable alternative therapies [21]. Several reviews of herbal extracts and secondary metabolites are used in conventional medicine owing to their anxiolytic and antidepressant properties [22,23]. Clinical and pre-clinical trials have reported successful use of medications for certain plant metabolites, such as alkaloids, terpenes, flavonoids, and sterols and others [24,25]. Medicinal plants have also proven efficacy against depression and anxiety, reported with the use of multiple plants, such as *Carum carvi* polyphenols at doses of 50 and 100 mg/kg [26], *Coriandrum sativum* at doses of 50, 100, and 200 mg/kg [27], *Origanum majorana* polyphenols at doses of 50 and 100 mg/kg [28], and *Petroselinum sativum* at doses of 50 and 100 mg/kg [29]. 

In this research, various animal models of anxiety and depression were used to demonstrate the antidepressant and anxiolytic effects of PATE, and also the memory impact of the subacute treatment, as this is the most frightening side effect of conventional drugs. The antidepressant effect of the PATE was tested in the FST and in TST. Both models placed animals in an unavoidable position and they became immobile, resembling a state in which behavioral despair imitates human depression [30,31]. As the dependent variable (immobility) is a direct reaction to the test and does not persist outside of the test environment, the forced swim and tail suspension procedures are best considered as basic antidepressant tests rather than models of depression [32]. Although there are construct validity components, there is no clear induction of a “depressive state” (stressful inducing conditions, decreased behavioral output). The learned helplessness technique, in which earlier exposure to unpleasant stress causes a more long-lasting alteration in which animals are subsequently less able to acquire appropriate escape reactions, is more similar to a depression model [33,34]. Nonetheless, the techniques described above have been utilized not only to evaluate the potential antidepressant efficacy of test drugs, but also to investigate putative neurological substrates of depression [34,35,36].

In FST, PATE significantly shortened the duration of immobility at both doses (100 and 200 mg/kg). This decrease was greater than that seen in mice treated with paroxetine. Likewise, in TST, a decrease in the downtime was observed in treated mice with 100 and 200 mg/kg of PATE. These results suggest that the total extract of *P. anisum* has potential as an antidepressant agent.

Two classical models of anxiety-like behavior tests were conducted to assess the anxiolytic activity of PATE, namely, LDBT and OFT. These are ethologically relevant assays for determining approach vs. avoidance behavior, baseline alertness, or defensive actions [37]. Approach–avoidant paradigms take advantage of circumstances in which environmental stimuli are seen as potentially threatening; delay to approach or time spent with a novel item (a possible threat) is assessed and utilized as a putative indication of anxiety. Rodents prefer dark and confined areas, which minimizes their vulnerability to predators [38].

For the LDBT, the brightness of the lighted compartment makes rodents feel anxious to explore this new environment. The time spent in the lighted compartment is considered an index of avoidance [39,40]. Since the PATE extract increases the crossing time of the lighted compartment, this fact suggests that it would abolish the avoidance behavior in the brightness of the illuminated space. In the OFT, sub-acute administration of PATE at a dose of 200 mg/kg exhibited a significant anxiolytic effect, even equally potent with that seen in the positive control group treated with bromazepam. The time spent at the center of the open field increased remarkably in a dose-dependent manner. Likewise, the number of tiles traversed during the open-field test increased significantly at the lowest dose used in this study. These results are in accordance with other studies using OFT to investigate the anxiolysis effect of plants and other drugs [26].

Registered with his name in the early 1980s, professor Morris developed the MWMT as a general assay of cognitive function, spatial learning, and memory [41] by placing animals in a large circular pool of water and escaping from it onto a hidden platform whose location can normally be identified only using spatial memory. Additionally, following the same principle on memory testing and assessing cognitive function, the Novel Object Recognition Test tests the ability of the mice to recognize new and familiar objects by calculating the amount of time taken to explore the new object, and that provides an index of recognition memory [42].

After an acute daily administration (21 days) and based on the data presented, PATE did not alter memory during MWMT or the NORT compared to the changes seen with the conventional drug “bromazepam”, which clearly altered the mice memory function during the tests, confirming the documented side effect.

Overall, our findings suggest that PATE has an anxiolytic and antidepressant-like activity and also prevents the memory side effect presented by conventional drugs. 

The observed results seem to be mediated by the active component present in the extract, such as phenolic acids, flavonoids, and others (Gallic acid, catechin, chlorogenic acid, caffeic acid, oleuropein, *p*-coumaric acid, trans-4-hydroxy-3-methoxycinnamic acid, myricetin, quercetin). The different parts of medicinal plants have various active molecules belonging to different families that are renowned for their various therapeutic activities, mainly flavonoids, which are characterized by their antidepressant and anxiolytic properties. Previous studies have demonstrated the antidepressant and anxiolytic effects of some of the identified bioactive components. Lee et al. have demonstrated that *p*-coumaric acid has improved LPS-induced despair-related symptoms by preventing the increase of inflammatory cytokines in the hippocampus and the reduction of brain-derived neurotrophic factor (BDNF) [43]. In a study conducted by Khan et al., it was found that quercetin’s antidepressant-like effect is mediated by its reduced excitotoxicity and augmented serotonin levels [44], while another study suggests that *n*-methyl-d-aspartate (NMDA) receptors are involved in the anxiety-like properties of caffeic acid in mice [45]. Additionally, it was found that catechins could inhibit monoamine oxidase (MAO), which may contribute to overall demonstrated activity by the anise extract [46]. The anti-depressive and anxiolytic effects of other bioactive components identified in this plant were also investigated in multiple studies [25,43,47,48,49,50,51,52,53,54,55,56].

## 4. Materials and Methods

### 4.1. Plant Material

*P. anisum* L. seeds were purchased in January 2020 from a local herbalist. They were identified and authenticated by professor Amina Bari (botanist), and a voucher specimen (BPRN73) was deposited in the herbarium of the Laboratory of Biotechnology, Environment, Agri-food, and Health, in the Faculty of Sciences Dhar el Mahraz, Fez, Morocco.

### 4.2. PATE Preparation

In a 500 mL bottle, 20 g of *P. anisum* powder was mixed with 200 mL of hexane and placed in a sonicator bath at a frequency of 35 kHz for 45 min at 25 °C to get rid of the oils contained in the powder (defatting).

The defatted powder was re-extracted with a 70% hydro-ethanolic solution (70%) under the same conditions as the first extraction. After filtration and drying, the residue was collected and stored at 4 °C until further usage.

### 4.3. HPLC Analysis

PATE was analyzed using high-performance liquid chromatography (Agilent Technologies 1260 infinity II). The apparatus was outfitted with a quaternary pump and a UV detector. The C18 zorbax eclipse plus C18 (5 μm, 4.6 × 150 mm) was the column that was used for the separation. Two solvents, A: water:acetic acid (95:5) and B: acetonitrile, constituted the mobile phase. The sample volume injection was 10 μL, and the flow rate was 1 mL/min. The analysis time was 65 min and the gradient adopted was 0–40 min: 50% A, 50% B; 40–45 min: 40%A, 60% B; 45–60 min: 100% B; 60–65 min: 96% A and 4%B. UV absorbance was monitored from 200 to 400 nm.

### 4.4. Animals

The animal house at the FSDM, Fez, was supplied with 221 Swiss albino mice with weights ranging between 25 and 35 g for this study. For two weeks (an adaptation time), they were put into groups of 5 in standard cages before being used in various experiments, a time during which the animals had free access to food and water and were held at a steady temperature of 24 ± 2 °C and a 12/12 h light/dark cycle.

**Figure 9:**
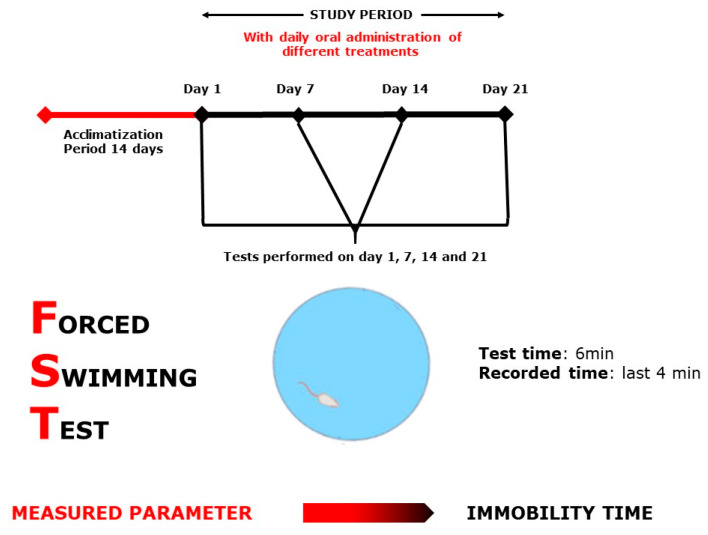
Forced Swimming Test scheme.

**Figure 10:**
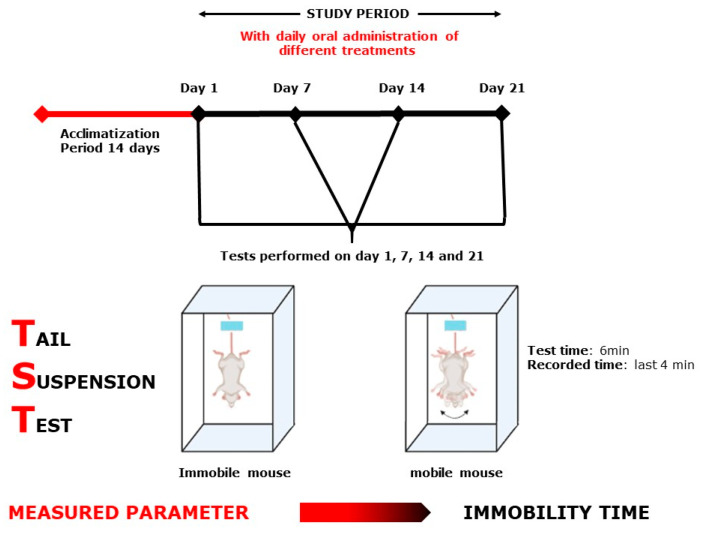
Tail Suspension Test scheme.

**Figure 11:**
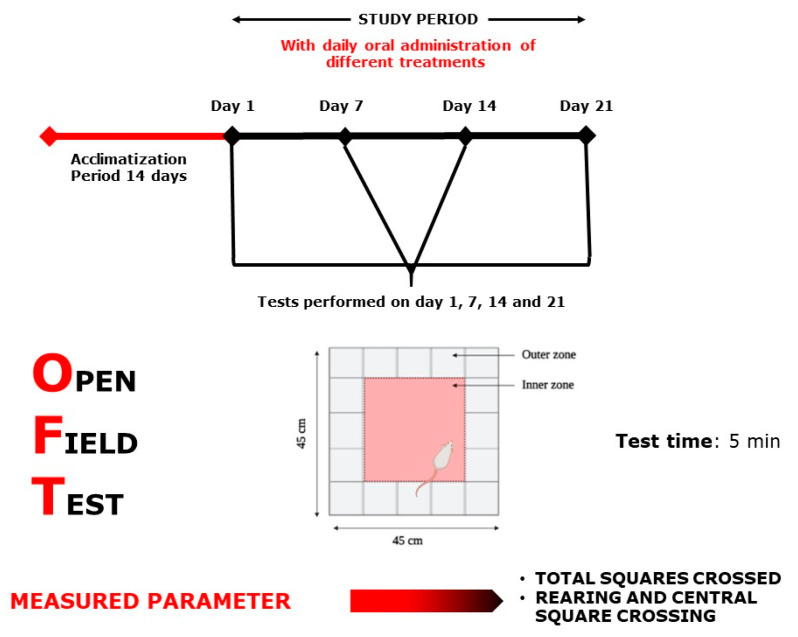
Open Field Test scheme.

**Figure 12:**
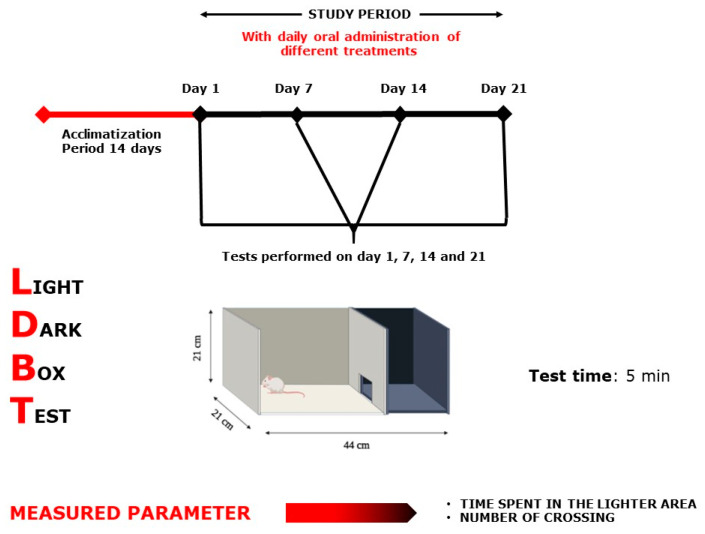
Light–Dark Box Test scheme.

**Figure 13:**
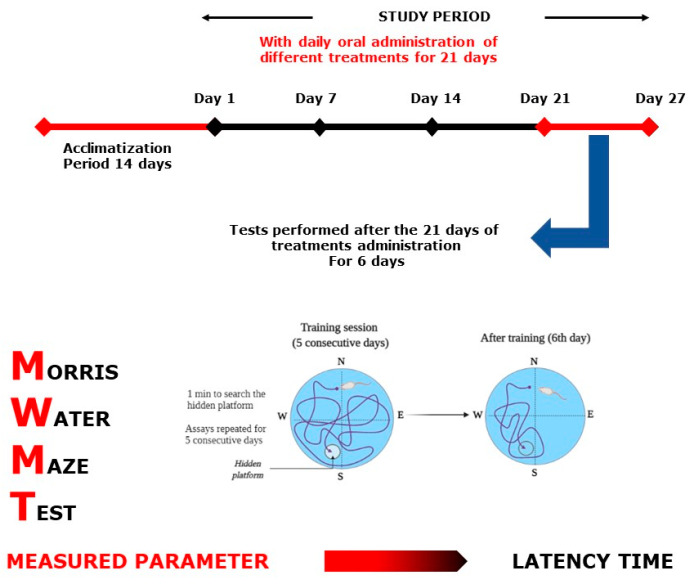
Morris Water Maze Test scheme.

**Figure 14:**
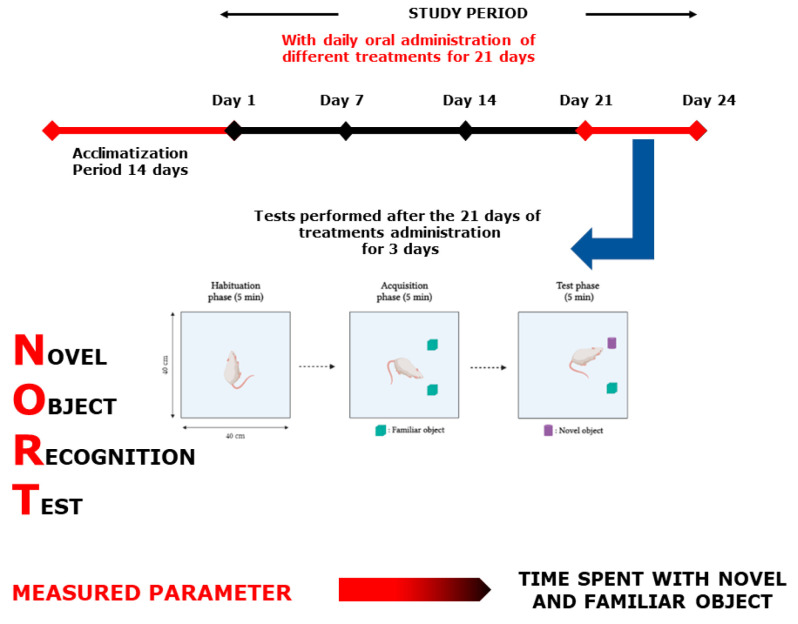
Novel Object Recognition Test scheme.

Each test group was designed separately with *n* = 7–8/treatment. As positive controls, bromazepam (1 mg/kg) for anxiety tests and paroxetine (11.5 mg/kg) for the depression tests were used. All 32 selected groups were treated sub-acutely for 21 days and tested on days 1, 7, 14, and 21, except for the MWM test, where the mice were treated continuously for 21 days and tested during the following six days, and the NORT, where the mice were treated for 21 days and tested during the following 3 days (Figure 9, Figure 10, Figure 11, Figure 12, Figure 13 and Figure 14). The animals were transferred from their cages to the experimental room and were habituated for at least one hour prior to treatment administration and the start of the behavioral tests. Treatments (PATE and drugs) were administered daily and orally to the animals by intragastric gavage using a stainless-steel gavage needle with a pear-shaped tip to introduce them into the stomach. All treatments were given 1 h prior to testing.

Each administered treatment was prepared by mixing the solid selected dose (e.g., 100 mg, 200 mg, etc.) with distilled water to fill a 10 mL tube. Based on the animal weight, and taking into consideration that the prepared solution of 100 mg/10 mL stands for 1 kg/1000 g, for each mouse, the amount of solution given was calculated using a simple rule of 3. As for the control group, they received 0.2 mL of distillated water daily (the solution in which the treatment was dissolved).

The doses of paroxetine and bromazepam were calculated based on the Food Drug Administration (FDA) guidance on human and animal dose conversion (media: 72309). The commonly used human doses of paroxetine and bromazepam were converted to those of the mice (paroxetine 11.5 mg/kg and bromazepam 1 mg/kg). As for the PATE doses, preliminary tests were conducted to select the lowest effective doses for the study up to 500 mg/kg to finally select the doses of 100 and 200 mg/kg. Care and handling of animals have been carried out in accordance with internationally accepted standards for the use of animals [57], and the institutional committee approved the study (12/2019/LBEAS).

### 4.5. Antidepression-Like Tests

#### 4.5.1. Forced Swimming Test

The FST (Figure 9), by [58], was used as described in [26]. Mice were placed singly and forced to swim in a rectangular pool with the following dimensions (50 × 30 × 60), filled with water to 25 cm, maintained at 28–29 °C. The test is based on the idea that the animal would deliberately swim to avoid stressful stimulation. The total period of immobility was observed during 4 min of the 6 min total test duration.

#### 4.5.2. Tail Suspension Test

The TST (Figure 10) is a mouse behavioral test that can be used to screen new antidepressant medicines, as well as to determine other manipulations that are likely to influence depression-related behaviors. During this test, the animal was hung by the tail (2 cm away from the end of the box) for 6 min; only the last 4 min was recorded.

### 4.6. Anxiolytic Assessment Tests

#### 4.6.1. Open Field Test

This experiment was carried out in a wooden apparatus (Figure 11) divided into 25 equal squares (45 × 45 × 13 cm). The sum of ambulation (the sum of total squares crossed), rearing, and central square crossings was captured using a digital video camera after placing each mouse in the center square as a starting point [59,60].

#### 4.6.2. Light–Dark Box Test 

The instrument was made up of two wooden boxes (44 × 21 × 21 cm), one dark and one white (Figure 12). A wooden blank separated the two compartments, with a 7 × 7 cm hole in the center on the ground surface. A 100 W lamp was mounted at 30 cm, well above the white box’s surface (the only light source in the room). At 5 min, the mice were examined [61]. The amount of time spent in the light zone, as well as the number of crossings between two compartments was examined [62]. After each use, the equipment was washed to remove any odors or leftovers that could annoy the mouse in the next trial.

### 4.7. Memory Assessment Tests

#### 4.7.1. Morris Water Maze Test

MWMT (Figure 13) is among the most commonly used cognitive neuroscience procedures for the study of spatial perception and memory psychological processes and neuronal pathways. In the pool, water was maintained at 24–25 °C. Every mouse was released from one of four directions of training (north, east, south, and west) on the surface of the water and given 1 min to swim to search for a hidden platform. A video camera was used to record the time latency (to find the hidden platform). The experimenter conducted the mice who could not find the platform and allowed them 10 s to remain on the platform [63]. Assays were replicated each day for 5 consecutive days. Then, on the sixth day, the platform was removed, and mice were allowed to swim for one minute, and the time spent swimming plus the distance traveled in the target quadrant was recorded.

#### 4.7.2. Novel Object Recognition Test

This test is focused on rodents’ random trend of exploring a novel object for more time than a common one. It is commonly used to evaluate memory in rodents. It was conducted in an open square box (Figure 14). The task procedure consists of three phases: the habituation, acquisition, and test phase. In the first session, the mice were allowed to explore in the open-field box, where they were put for 5 min, 10 cm from the sidewall. In the second session, the mice were allowed to discover two similar objects (familiar object (F + F)) after placing the mouse in the center of the box. The last session took place when one of the two familiar objects was changed to a novel one (N) for 5 min, similar to the other sessions. Turning or standing on the object was not seen as a behavior of exploration, the nose should be directed to the target at a distance of no further than 2 cm, or the object should be touched by the nose. During the 1st and 2nd assessments, time was spent exploring each object [64]. The results of this test were presented as a discrimination index calculated as follows:

DI = (N − F)/(N + F)

N = time spent in the novel object; F = time spent in the familiar object.

## 5. Conclusions

Thus, our results provide pieces of evidence that PATE possesses strong antidepressant and anxiolytic activities with no effect on memory, through multiple tests, with a possible monoaminergic involvement in the mode of action of the extract based on the studied mode of action of some molecules contained in the extract. Anise seeds are considered a safe plant, belonging to the category of traditional, well-documented medicinal plants in the European pharmacopeia (EMEA/HMPC/137421/2006). In view of these findings, this plant presents a potent potential that could be well established as a completely safe alternative to conventional drugs or to be recommended as a complement.

## Figures and Tables

**Table 1:** Chemical composition of the PATE.

Compounds	RT (Retention Time)	Concentration (μg/g)
Gallic acid	6.652	4.17
Catechin	11.097	11.66
Chlorogenic acid	17.555	2.78
Caffeic acid	18.28	3.7
Oleuropein	21.03	8.6
*p*-coumaric acid	21.648	16.4
Trans-4-hydroxy-3-methoxycinnamic acid	22.097	0.8
Myricetin	22.381	0.06
Quercetin	23.224	0.35

## Data Availability

Data are available upon reasonable request.

## Supplementary Materials

The following are available online at https://www.mdpi.com/article/10.3390/plants10081573/s1, Figure S1: PATE HPLC Chromatogram.
