# Supplementary figures and images for: Assessment of Antidepressant-like, Anxiolytic Effects and Impact on Memory of Pimpinella anisum L. Total Extract on Swiss Albino Mice
Source: Plants (Basel). 2021 Jul 30;10(8):1573. doi: 10.3390/plants10081573 (PMC8399924; doi:10.3390/plants10081573)

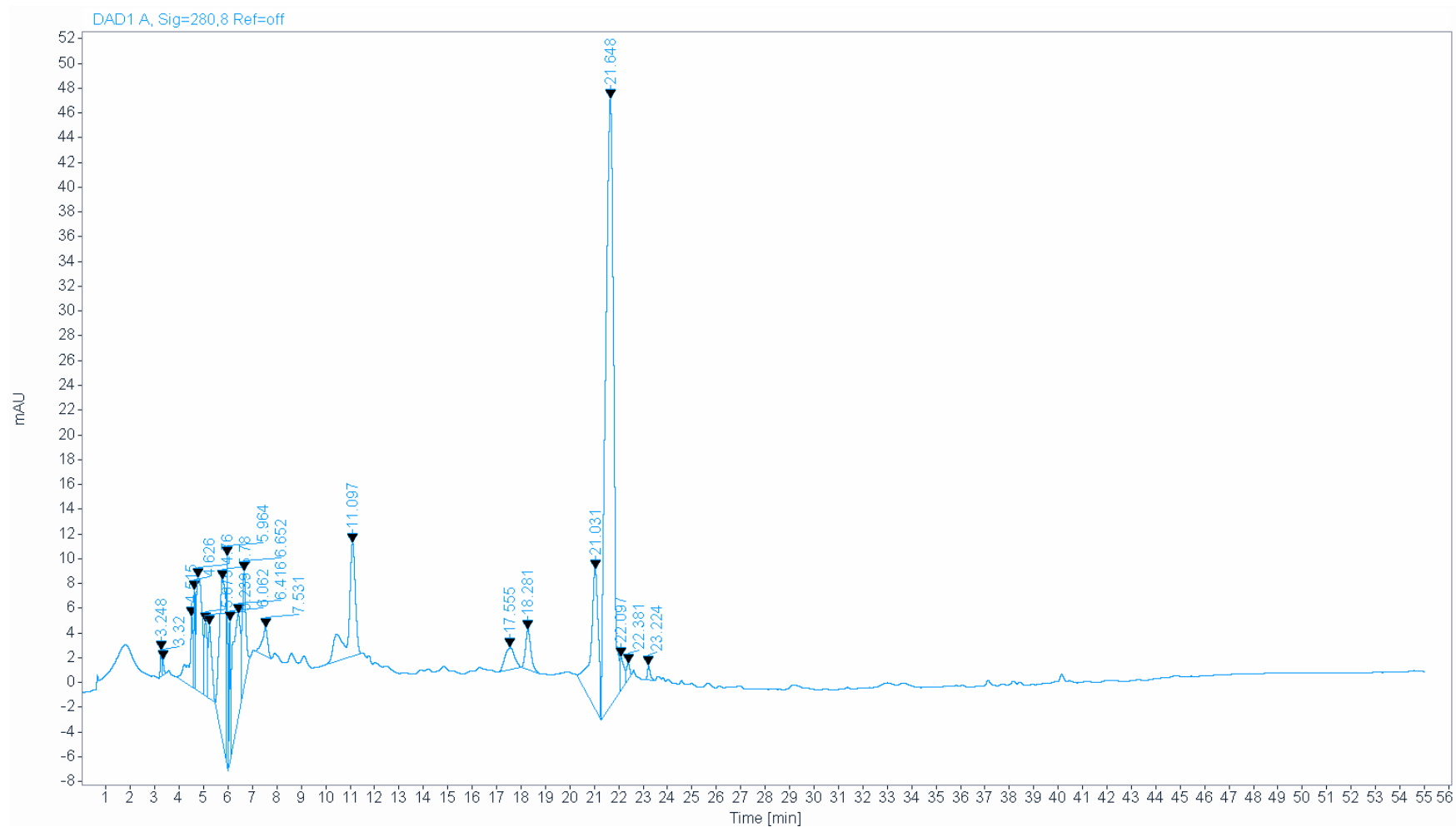

Supplement: Supplementary file 1 [file plants-10-01573-s001.zip › plants-1286312-supplementary.pdf]
